# Supplementary material for: Why can people with developmental prosopagnosia recognise some familiar faces? Insights from subjective experience
Source: PeerJ. 2023 Jul 18;11:e15497. doi: 10.7717/peerj.15497 (PMC10361072; doi:10.7717/peerj.15497)
Supplement: Supplemental Information 1 — Note. To be included in the DP sample, participants were required to self-report severe difficulties in everyday face recognition and to show impaired performance (at least 1.7 SDs from the control mean, e.g., DeGutis, Cohan & Nakayama, 2014; Murray et al., 2021) on at least two of three objective screening tasks (for recent discussion about the benefits of using more liberal inclusion criteria in the diagnosis of DP see Burns et al., 2022; DeGutis et al., 2023). Participants also scored within the typical range on the Autism Quotient (Baron-Cohen et al., 2001). 1 The Cambridge Face Memory Test (Duchaine & Nakayama, 2006); 1.7SD cut-off score of 46/72 (norms from the age-matched controls of Duchaine, Yovel & Nakayama, 2007). 2 The Cambridge Face Perception Test (Duchaine, Germine & Nakayama, 2007); 1.7SD cut-off score of 58 (norms from the age-matched controls of (Duchaine, Germine & Nakayama, 2007)). Note that scores represent the number of errors, such that higher values indicate poorer performance. 3 Famous Face Test (Bate et al., 2019b); 1.7SD cut-off score of 80% (norms from the age-matched controls of Bate et al., 2019b). [file peerj-11-15497-s001.docx]

**Supplemental Table 1:**

**Screening scores for all DP participants**

| Participant ID | CFMT^1^ | CFPT^2^ | Famous^3^ |
| --- | --- | --- | --- |
| P1 | 40 | 52 | 47.37 |
| P2 | 33 | 48 | 38.89 |
| P3 | 44 | 46 | 40.43 |
| P4 | 32 | 66 | 46.43 |
| P5 | 39 | 62 | 67.30 |
| P6 | 32 | 80 | 32.14 |
| P7 | 42 | 66 | 21.05 |
| P8 | 33 | 54 | 55.93 |
| P9 | 36 | 58 | 31.67 |
| P10 | 42 | 66 | 65.91 |
| P11 | 43 | 44 | 28.33 |
| P12 | 33 | 64 | 47.83 |
| P13 | 41 | Not completed | 40.00 |
| P14 | 45 | 60 | 43.86 |
| P15 | 35 | 70 | 25.00 |
| P16 | 35 | 80 | 49.02 |
| P17 | 31 | 48 | 31.48 |
| P18 | 36 | 78 | 57.89 |
| P19 | 38 | 66 | Not completed |
| P20 | 46 | 38 | 15.52 |
| P21 | 45 | 78 | 79.00 |
| P22 | 42 | 52 | 45.83 |
| P23 | 41 | 66 | 80.36 |

*Note*. To be included in the DP sample, participants were required to self-report severe difficulties in everyday face recognition and to show impaired performance (at least 1.7 SDs from the control mean, e.g., DeGutis et al., 2014, Murray et al., 2021) on at least two of three objective screening tasks (for recent discussion about the benefits of using more liberal inclusion criteria in the diagnosis of DP see Burns et al., 2022; DeGutis et al., 2023). Participants also scored within the typical range on the Autism Quotient (Baron-Cohen et al., 2001).

^1^ The Cambridge Face Memory Test (Duchaine & Nakayama, 2006); 1.7SD cut-off score of 46/72 (norms from the age-matched controls of Duchaine, Yovel & Nakayama, 2007).

^2^ The Cambridge Face Perception Test (Duchaine, Germine & Nakayama, 2007); 1.7SD cut-off score of 58 (norms from the age-matched controls of Duchaine, Germine & Nakayama, 2007). Note that scores represent the number of errors, such that higher values indicate poorer performance.

^3^ Famous Face Test (Bate et al., 2019b); 1.7SD cut-off score of 80% (norms from the age-matched controls of Bate et al., 2019b).

References

Baron-Cohen, S., Wheelwright, S., Skinner, R., Martin, J., & Clubley, E. (2001). The Autism-Spectrum Quotient (AQ): Evidence from Asperger Syndrome/High-Functioning Autism, males and females, scientists and mathematicians. *Journal of Autism and Developmental Disorders,* 31: 5-17. https://doi.org/10.1023/A:1005653411471

Bate, S., Bennetts, R., Gregory, N.J., Tree, J., Murray, E., Adams, A., … Banissy, M. (2019b). Objective patterns of face recognition deficits in 165 adults with self-reported developmental prosopagnosia. *Brain Sciences,* 9: 133. https://doi.org/10.3390/brainsci9060133.

Burns, E.J., Gaunt, E., Kidane, B., Hunter, L., & Pulford, J. (2022). A new approach to diagnosing and researching developmental prosopagnosia: Excluded cases are impaired too. *Behavior Research Methods*. https://doi.org/10.3758/s13428-022-02017-w.

DeGutis, J., Bahierathan, K., Barahona, K., Lee, E., Evans, T.C., Shin, H.M., … Wilmer, J.B. (2023). What is the prevalence of developmental prosopagnosia? An empirical assessment of different diagnostic cutoffs. *Cortex,* 161: 51-64. https://doi.org/10.1016/j.cortex.2022.12.014

DeGutis, J., Cohan, S., & Nakayama, K. (2014). Holistic face training enhances face processing in developmental prosopagnosia. *Brain,* 137: 1781-1798. https://doi.org/10.1093/brain/awu062

Duchaine, B.C., Germine, L., & Nakayama, K. (2007). Family resemblance: Ten family members with prosopagnosia and within-class object agnosia. *Cognitive Neuropsychology,* 24(4): 419-430. https://doi.org/10.1080/02643290701380491

Duchaine, B.C., & Nakayama, K. (2006). The Cambridge face memory test: Results for neurologically intact individuals and an investigation of its validity using inverted face stimuli and prosopagnosic participants. *Neuropsychologia,* 44(4): 576-585. https://doi.org/10.1016/j.neuropsychologia.2005.07.001

Duchaine, B.C., Yovel, G., & Nakayama, K. (2007). No global processing deficit in the Navon task in 14 developmental prosopagnosics. *Social Cognitive and Affective Neuroscience,* 2: 104-113. https://doi.org/10.1093/scan/nsm003

Murray, E., Bennetts, R., Tree, J., & Bate, S. (2022). An update of the Benton Facial Recognition Test. *Behavior Research Methods,* 54(5): 2318-2333. https://doi.org/10.3758/s13428-021-01727-x.
