# Supplementary material for: Why can people with developmental prosopagnosia recognise some familiar faces? Insights from subjective experience
Source: PeerJ. 2023 Jul 18;11:e15497. doi: 10.7717/peerj.15497 (PMC10361072; doi:10.7717/peerj.15497)
Supplement: Supplemental Information 4 [file peerj-11-15497-s004.pdf]

Supplemental Article 1: Study 1 face recognition questionnaire and list of celebrity target identities

Section 1: Compensated Recognition

Participants Instructions: This section enquires about your ability to recognise familiar people using only compensatory cues to identity. This refers to any strategies that you use to "work out" who the person is, such as a distinguishing facial feature, their hairstyle, context, or the way that they walk. It is not referring to the "instant" or "automatic" recognition that you might imagine people without face blindness are able to use.

- **Q1 [Displayed to all participants]:** Are you able to successfully recognise faces using compensatory cues to identity (e.g. your recognition of the faces is not "automatic" but you are able to work out who the person is using cues such as context, hairstyle or accessories)?
  - **Response Options:**
    1. I can recognise the faces of ALL the people that I know using compensatory cues
    2. I can recognise the faces of MANY of the people that I know using compensatory cues
    3. I can recognise the faces of SOME of the people that I know using compensatory cues
    4. I can recognise the faces of FEW of the people that I know using compensatory cues
    5. I can recognise the faces of NONE of the people that I know using compensatory cues

- **Q2 [Displayed only if the participant selected options 1 – 4 are selected for Q1]:** For those individuals that you can recognise using compensatory cues, are these cues successful all of the time?
  - **Response Options:**
    1. I ALWAYS recognise particular people without making errors
    2. I FREQUENTLY recognise particular people without making errors
    3. I SOMETIMES recognise particular people without making errors
    4. I RARELY recognise particular people without making errors
    5. I NEVER recognise particular people without making errors

- **Q3 [Displayed only if the participant selected options 2 – 5 for Q2]:** Why do you think that your strategies sometimes fail?
  - [Open-ended response invited]

- **Q4 [Displayed only if the participant selected options 1 – 4 for Q1]:** Does your ability to successfully use compensatory cues decay if you do not regularly see that person?
  - **Response Options:**
    1. Always
    2. Often
    3. Sometimes
    4. Rarely
    5. Never

- **Q5 [Displayed to all participants]:** Have you ever mistakenly thought that an unknown person is familiar when using compensatory cues to identity?
  - **Response Options:**
    1. Very Frequently
    2. Frequently
    3. Occasionally
    4. Rarely
    5. Never

- **Q6 [Displayed to participants who selected options 1 – 4 to Q5]:** Why do you think these errors occurred?
  - [Open-ended response invited]

Section 2: Spontaneous Recognition

- **Q1 [Displayed to all participants]:** Have you ever spontaneously or automatically recognised a face without the need to apply compensatory or contextual cues (i.e. have you recognised a face in the same way as you might imagine a person without face recognition difficulties can, by "instantly knowing" who that person is from their face, without having to "work it out")?
  - **Response Options:**
    1. Very Frequently
    2. Frequently
    3. Occasionally
    4. Rarely
    5. Never

- **Q2 [Displayed only to participants who selected options 1 – 4 to Q1]:** For those individuals that you have previously managed to spontaneously recognise, can you successfully do this every time you see those particular faces?
  - **Response Options:**
    1. Always

2. Often
3. Sometimes
4. Rarely
5. Never

- **Q3 [Displayed only to participants who selected options 1 – 4 in response to Q2]:** Would you say that your spontaneous/automatic recognition of these people occurs as quickly as it does for people without face recognition difficulties?
  - **Response Options:**
  1. Always
  2. Often
  3. Sometimes
  4. Rarely
  5. Never
  6. Don't Know

- **Q4 [Displayed only to participants who selected options 1 – 4 in response to Q1]:** Does your ability to spontaneously recognise a face decay if you do not regularly see that person?
  - **Response Options:**
  1. Always
  2. Often
  3. Sometimes
  4. Rarely
  5. Never

- **Q5:** Have you ever spontaneously recognised someone in error (i.e. the person didn't turn out to be the person that you thought it was)?
  - **Response Options:**
  1. Very Frequently
  2. Frequently
  3. Occasionally
  4. Rarely
  5. Never

- **Q6 [Displayed only to participants who selected options 1 – 4 in response to Q5):** Why do you think these errors occurred?
  - **[Open-ended response invited]**

Section 3: Celebrity Familiarity Ratings

Participant Instructions: Earlier in this study we asked you to complete a face recognition test that displayed some images of celebrities. To ensure the integrity of our data, we need to establish whether any recognition errors result from your difficulties with faces, or occurred simply because you have no or little knowledge of that person per se (e.g. if you are not interested in sports or movies, you would be unlikely to recognise certain sports stars or actors even if you had excellent face recognition skills).

Please rate your overall familiarity with each celebrity (i.e. your awareness of the person regardless of your ability to recognise their face) in the table below. The celebrities contained in the study were ranked as the 100 most famous people in the UK in a recent YouGov survey. We therefore expect that most people are familiar with the majority of the personalities and will respond at the top end of the scale for most faces. There are four screens to complete, each containing 25 names.

- **Celebrity Identities (N = 100):** Leonardo DiCaprio; David Beckham; Boris Johnson; Donald Trump; Gordon Ramsay; JK Rowling; Prince Harry, Brad Pitt; Prince Charles; Jamie Oliver; David Cameron; Robbie Williams; Britney Spears, Jeremy Corbyn; Madonna; Jeremy Clarkson; Simon Cowell; Nigella Lawson; Justin Bieber; Victoria Beckham; Ed Sheeran; Rowan Atkinson; Kim Kardashian; Jennifer Lopez; Tom Cruise; Arnold Schwarzenegger; Kylie Minogue; David Attenborough; George Clooney; Stephen Fry; Teresa May; Graham Norton; Angelina Jolie; Tom Hanks; Prince William; John Travolta; Nigel Farage; Hillary Clinton; Piers Morgan; Jennifer Anniston; Paul McCartney; Justin Timberlake; Jeremy Kyle; George W Bush; Hugh Grant; Sylvester Stallone; Mariah Carey; Prince Philip; Liam Gallagher; Mick Jagger; Tom Jones; Johnathan Ross; Daniel Craig; Julia Roberts; Bruce Willis; Tony Blair; Dolly Parton; Robert DeNiro; Phillip Schofield; Cher; Russell Brand; Richard Branson; Celine Dion; Judi Dench; Rod Stewart; Taylor Swift; Christina Aguilera; Cheryl Cole; Gordon Brown; Michael McIntyre; Kate Winslet; Cameron Diaz; Sharon Osbourne; Sandra Bullock; Daniel Radcliffe; Kate Moss; Jimmy Carr; Bear Grylls; Megan Markle; Michael Buble; Alan Sugar; Princess Anne; Ariana Grande; Nicolas Cage; Katy Perry; Mary Berry; Ed Miliband; Bill Clinton; Katie Price; David Tennant; Pamela Anderson; Ricky Gervais; Wayne Rooney; Meryl Streep; Nicole Kidman; David Hasselhoff; Gary Lineker; Pierce Brosnan; Andy Murray; Harrison Ford.

- **Familiarity Response Options, per celebrity name:**
  1. Not at all Familiar
  2. A little Familiar
  3. Somewhat Familiar
  4. Familiar
  5. Very Familiar
